# Сахарный диабет и онкопатология: система инсулиноподобных факторов роста

**DOI:** 10.14341/probl12741

**Published:** 2021-09-10

**Authors:** Е. М. Франциянц, Е. И. Сурикова, И. В. Каплиева, В. A. Бандовкина, И. В. Нескубина, Е. А. Шейко, М. И. Морозова, И. М. Котиева

**Affiliations:** Национальный медицинский исследовательский центр онкологии; Национальный медицинский исследовательский центр онкологии; Национальный медицинский исследовательский центр онкологии; Национальный медицинский исследовательский центр онкологии; Национальный медицинский исследовательский центр онкологии; Национальный медицинский исследовательский центр онкологии; Национальный медицинский исследовательский центр онкологии; Национальный медицинский исследовательский центр онкологии

**Keywords:** сахарный диабет, онкопатология, злокачественные опухоли, система инсулиноподобных факторов роста

## Abstract

Сахарный диабет и злокачественные опухоли — одни из самых частых и сложных заболеваний. Эпидемиологические исследования показали наличие сильной взаимосвязи между этими патологиями. Причинность этой связи до сих пор однозначно не установлена, но предложен ряд вероятных биологических механизмов, объясняющих ее через эффекты гипергликемии, гиперинсулинемии на процесс онкогенеза. Важную роль при этом играет ось инсулиноподобных факторов роста, их рецепторов и связывающих белков (IGF/IGFR/IGFBP). В обзоре приведены данные о структурных элементах сигнальной оси инсулин/IGF/IGFR/IGFBP и их внутренних взаимосвязях при сахарном диабете и при развитии злокачественных опухолей. Значительные изменения в оси, происходящие при формировании диабетической среды, подготавливают фон, который при определенных условиях может обусловить стимуляцию или ингибирование развития опухоли. Рассмотренная сигнальная система, играя значительную роль в физиологии нормальных клеток, зачастую функционирует как решающий фактор выживания опухолевых клеток, обеспечивая тонкую контекстно-зависимую регуляцию многих клеточных процессов, связанных с онкогенезом. Однако, несмотря на многолетние глубокие исследования патогенеза сахарного диабета и злокачественных опухолей, молекулярные механизмы взаимосвязи этих патологий еще в значительной степени неясны, а внутренняя неоднородность патологий усложняет проведение исследований и интерпретацию результатов, оставляя множество вопросов.

## Abstract (en)

Diabetes mellitus and malignant tumors are among the most common and complex diseases. Epidemiological studies have shown a strong relationship between these pathologies. The causality of this relationship has not yet been unambiguously established, but a number of probable biological mechanisms have been proposed to explain it through the effects of hyperglycemia, hyperinsulinemia on the process of oncogenesis. An important role in this is played by the axis of insulin-like growth factors, their receptors and binding proteins (IGF / IGFR / IGFBP). The review provides data on the structural elements of the insulin / IGF / IGFR / IGFBP signaling axis and their internal relationships in diabetes mellitus and in the development of malignant tumors. Significant changes in the axis that occur during the formation of the diabetic environment prepare the background, which, under certain conditions, can lead to the stimulation or inhibition of tumor development. The considered signaling system, playing a significant role in the physiology of normal cells, often functions as a decisive factor in the survival of tumor cells, providing fine context-dependent regulation of many cellular processes associated with oncogenesis. However, despite many years of in-depth studies of the pathogenesis of diabetes mellitus and malignant tumors, the molecular mechanisms of the relationship between these pathologies are still largely unclear, and the internal heterogeneity of pathologies complicates research and interpretation of the results, leaving many questions.

## ВВЕДЕНИЕ

Онкологическая патология и сахарный диабет находятся в ряду наиболее распространенных патологий человека, заболеваемость которыми увеличивается год от года. В мире сахарным диабетом (СД) страдают 463 млн человек в возрасте 20–79 лет, еще около 232 млн человек не знают, что у них есть это заболевание, поэтому не получают соответствующего лечения и подвергаются высокому риску развития осложнений, связанных с диабетом. Почти 374 млн человек имеют нарушение толерантности к глюкозе [1]. В Российской Федерации общая численность пациентов с СД на 01.01.2019 г. составила 4 584 575 (3,12% населения РФ), при этом доля невыявленного СД2 в РФ в среднем составляет 54% [2]. При анализе данных федерального регистра СД выявлены наиболее важные тенденции — сохранение стабильного роста распространенности СД в РФ при подавляющем большинстве пациентов с СД2, что подтверждает глобальные мировые тенденции увеличения доли СД2 среди общего количества пациентов с СД [2].

Наряду с ростом СД во всем мире наблюдается аналогичная устойчивая тенденция роста онкологических заболеваний. По данным Всемирной организации здравоохранения, в 2018 г. в мире было зарегистрировано 18,1 млн случаев рака, а к 2040 г. эксперты прогнозируют рост заболеваемости до 29,5 млн случаев за год [3]. В России ежегодно регистрируется в среднем более 600 тыс. новых случаев онкопатологии, что составило в 2019 г. почти 4 млн человек (2,7% населения РФ) [4]. В России, как и во всем мире, онкологические заболевания, наряду с сердечно-сосудистыми, продолжают лидировать в списке болезней с высоким уровнем смертности: в 2020 г. в мире от онкопатологии умерли почти 10 млн человек [5], в России ежегодно умирают от онкологии в среднем около 300 тыс. человек, доля смертности от злокачественных новообразований в 2019 г. составила 16,4% [4].

Увеличение в целом выживаемости онкологических больных, связанное с повышением эффективности лечения в последнее десятилетие, привело к увеличению доли пациентов, живущих с двумя и более сопутствующими хроническими заболеваниями, среди которых СД является одним из самых распространенных. В связи с этим растет интерес к состоянию мультиморбидности, т.к. оно связано с худшими клиническими исходами, увеличенной смертностью, более высокими затратами на здравоохранение, при этом большинство пациентов с мультиморбидными состояниями находятся в трудоспособном возрасте [6][7].

Согласно данным о распространенности СД и рака, полученным из опубликованных метаанализов, в 2012 г. СД мог быть причиной примерно 280 100 (2,0%) случаев рака во всем мире [8]. У пациентов с СД заболеваемость раком в целом на 15–30% выше, чем у людей без диабета, и зависит от локализации опухоли [9][10].

Повышенный уровень глюкозы в крови, как было установлено, оказывает стимулирующее влияние на пролиферацию опухолевых клеток [11]. Обнаружена сильная корреляция между СД и канцерогенезом. ­Однако учитывая, что соотношение между СД 2 и 1 типа 10:1 и онкопатология в основном характерна для пожилых людей (среди которых СД 1 типа встречается значительно реже), можно предположить, что большинство опухолей наблюдается у пациентов с СД 2 типа (СД2), а связь между СД2 и онкопатологией сильнее по сравнению с СД 1 типа [8][12].

Эпидемиологические данные свидетельствуют о том, что люди с СД обоих типов имеют значительно более высокий риск развития некоторых форм рака (печени, поджелудочной железы, эндометрия, толстой и прямой кишки, молочной железы, мочевого пузыря) [13]. Во многих исследованиях сообщается, что при СД2 прежде всего значительно увеличивается риск заболевания раком поджелудочной железы — до 80% [14–16]. Причем обнаружено, что при меньшей длительности СД2 (менее 4–5 лет) риск злокачественного новообразования поджелудочной железы на 50% больше, чем у людей с бóльшей длительностью диабета [14]. В исследовании P.J. Hardefeldt и соавт. выявлен значительно больший риск рака молочной железы (РМЖ) у женщин с СД2, что поддерживает гипотезу о том, что СД является независимым фактором риска РМЖ [17]. При этом результаты о повышенном риске рака грудной ­железы у мужчин с СД не достигли уровня статистической значимости [17]. В ряде исследований была показана связь между риском СД и раком яичников, колоректальным раком, раком печени, почек [18–21]. В работе X. Wang и соавт. продемонстрирована взаимосвязь между СД и развитием и прогнозом опухолей головы и шеи [22]. Были выявлены половые особенности риска возникновения злокачественных опухолей у пациентов с СД [20][23]. Более того, была показана связь диабета и предиабета с повышенным риском смерти от рака, особенно от рака печени [10][24–26].

В исследовании X.H. Zhou и соавт. было установлено, что с увеличением концентрации глюкозы линейно возрастала смертность от злокачественных опухолей всех исследованных локализаций — рака желудка, прямой кишки, поджелудочной железы, печени, причем также имелись половые особенности [24]. Кроме того, данные ряда исследований показывают, что некоторые лекарственные препараты, используемые для лечения гипергликемии, связаны либо с повышенным, либо с пониженным риском развития злокачественных опухолей, особенно в популяции, уже подверженной онкологическому риску [15, 27].

В то же время имеются исследования, в которых связь между СД и повышенным риском злокачественных новообразований не подтверждается. Например, в работах Z.F. Miao и соавт., J. Zheng и соавт. не обнаружено увеличения риска рака желудка среди пациентов с предиабетом или СД [28][29]. В крупнейшем исследовании C.T. Rentsch и соавт. по изучению связи между заболеваемостью раком (16 локализаций) и уровнем гликозилированного гемоглобина с поправкой на широкий спектр факторов (демографических, социальных, клинических) не удалось выявить какой-либо независимой положительной связи между изучаемыми показателями за исключением рака поджелудочной железы. При этом неожиданно была показана обратная связь между уровнем гликозилированного гемоглобина и пременопаузальным РМЖ. На основании этих результатов авторы сомневаются в возможности прямой связи гипергликемии и риска злокачественных новообразований [30].

Таким образом, результаты большого числа исследований свидетельствуют о сложном характере связи между СД и риском развития злокачественных опухолей, зависящим от пола пациента, локализации, а значит, и биологических особенностей опухоли. Повышению смертности пациентов с СД и онкопатологией могут способствовать как общие факторы риска (возраст, пол, масса тела, диета, физическая активность, курение, алкоголь) и факторы, связанные с лечением СД, так и потенциальные биологические механизмы, которые пока изучены не полностью. Обе эти патологии относят к полиэтиологичным заболеваниям человека, в основе которых лежат как генетические изменения, так и нарушение функционирования регуляторной гормонально-метаболической сети организма [27]. В настоящий момент гипергликемия, гиперинсулинемия, воспаление и дисбаланс в системе половых гормонов являются основными гипотезами о возможных биологических механизмах, связывающих СД и онкопатологию [10][31][32].

Гипергликемия является основным характерным признаком всей группы метаболических заболеваний СД. При СД 1 типа (5–10% всех пациентов) гипергликемия обусловлена абсолютным дефицитом секреции эндогенного инсулина и абсолютной необходимостью введения экзогенного инсулина, что приводит к значительно более высокому уровню циркулирующего в крови инсулина, чем эндогенная его секреция. При СД2 (90–95% всех пациентов) гипергликемия из-за инсулинорезистентности периферических тканей сосуществует в течение длительного времени с гиперинсулинемией. В результате возникает дисбаланс регуляции в системе «глюкоза-инсулин-инсулиноподобные факторы роста (IGFs)-их рецепторы» [32][33].

## СТРУКТУРНЫЕ ЭЛЕМЕНТЫ СИГНАЛЬНОЙ СИСТЕМЫ ИНСУЛИНА И ОСИ IGF/IGFBP

Сигнальный путь инсулина и IGFs высококонсервативен среди многоклеточных животных. Регуляция метаболизма углеводов и поддержание физиологического уровня глюкозы в крови осуществляются прежде всего инсулином, обеспечивающим клеточное поглощение и использование глюкозы в тканях-мишенях (печень, мышцы, жировая ткань), но система IGFs также участвует в поддержании гомеостаза глюкозы и регуляции роста. В физиологических условиях гормон роста контролирует экспрессию инсулиноподобного фактора роста-1 (IGF-1) в клетках печени, а также локальную секрецию IGFs. Действуя аутокринно/паракринно, эти факторы роста опосредуют глобальные эффекты гормона роста. Инсулиноподобный фактор роста-2 (IGF-2) экспрессируется преимущественно пренатально, играя важную роль в росте плода, при этом у взрослого человека в отличие от IGF1 экспрессия IGF2 гепатоцитами не регулируется гормоном роста [34][35]. Дефицит IGF-1/-2 приводит к низкому росту, а избыток — к увеличению органов, наблюдаемому при акромегалии [36].

Помимо регуляции роста организма, IGFs участвуют во многих физиологических процессах, связанных с пролиферацией, дифференцировкой, выживанием, апоптозом клеток [37][38]. Метаболические и митогенные эффекты инсулина опосредуют его связывание с рецепторами (IR) на поверхности клеток-мишеней. Потеря чувствительности клеток-мишеней к физиологическому действию инсулина (инсулинорезистентность) приводит к нарушению клеточного поглощения глюкозы с ростом ее концентрации в крови (гипергликемия) и компенсаторному увеличению продукции инсулина β-клетками поджелудочной железы в попытке восстановить гомеостаз глюкозы. Избыточное производство инсулина и сопутствующее повышение его уровня в крови формируют состояние гиперинсулинемии. Высокий уровень инсулина стимулирует синтез IGF-1 в клетках печени [37].

Инсулин и IGFs, обладающие высокой степенью гомологии, связываются на мембране клеток-мишеней с рецепторами из семейства рецепторных тирозинкиназ (RTK), которые произошли от общего предкового гена и обладают высокой степенью гомологии [39]. Каждый рецептор инсулина IR представляет собой димер, существующий в двух изоформах — IR-A и IR-B. Экспрессия этих изоформ регулируется онтогенетически и тканеспецифична. Изоформа IR-A экспрессируется преимущественно в тканях эмбриона и плода, где она регулирует рост через передачу митогенных сигналов, изоформа IR-B — преимущественно в тканях-мишенях инсулина (в печени, жировой ткани и мышцах), регулируя его метаболические эффекты. Эти изоформы IR различаются функционально, что подчеркивается аномально высоким соотношением IR-A/IR-B в мышечных клетках пациентов с миотонической дистрофией, которое, по предположению авторов исследования, отвечает за инсулинорезистентность, наблюдаемую у этих пациентов [39][40].

Известно, что, кроме образования гомодимеров, рецепторы инсулина и IGF-1 могут образовывать гибридные рецепторы из изоформ IR-A, IR-B, IGF-1R (рецептор IGF-1), что приводит к сложной тканеспецифической регуляции метаболических и митогенных сигнальных путей инсулина и IGF [39][41]. Сложность рассматриваемой сети добавляет множество лигандов — инсулина, IGF-1, IGF-2, способных связываться с указанными выше рецепторами и различающихся сродством к каждому димеру рецептора. В настоящее время известно, что рецептор инсулина имеет в 100 раз большее сродство к инсулину, чем к IGF-1, сродство рецептора IGF-1R к IGF-1 в 1000 раз больше, чем к инсулину, изоформа IR-A связывает с высоким сродством не только инсулин, но также IGF-2 и проинсулин [37][41][42]. В физиологических условиях инсулин взаимодействует в первую очередь с рецепторами IR-A, IR-B и с гибридным IR-A/IR-B, а IGF-1 — с IGF-1R и с гибридами IR/IGF-1R. IGF-2 связывается с IGF-1R, с IR-A, а также с гибридными рецепторами IGF1R/IR-A, при этом его сродство к IR-B снижено, в связи с чем IGF-2 селективен в первую очередь в отношении изоформы IR-A. Кроме этого, IGF-2 также связывается с отдельным рецептором IGF-2R, который лишен домена тирозинкиназы и функционирует как рецептор-ловушка для ингибирования IGF-2-зависимой передачи сигналов через рецепторы IR и IGF-1R [35][39][41].

Взаимодействие инсулина и IGFs с рецепторами IR и IGF-1R инициирует каскад реакций фосфорилирования внутриклеточных белковых и липидных элементов, приводящий к регуляции различных сигнальных путей: в физиологических условиях воздействие на рецепторы IR-B и IGF1R/IR-В регулирует метаболические, а IR-А и IGF1R/IR-А — митогенные пути, воздействие на IGF1R в зависимости от контекста регулирует метаболические или митогенные пути сигнализации, проявляет мощную антиапоптотическую активность [41][43].

Первичными медиаторами инсулин-/IGF-зависимой регуляции метаболизма глюкозы и митогенеза в большинстве типов клеток являются белки IRS и Shc, активирующие соответственно пути фосфатидилинозитол-3-киназы (PI3K) и митогенактивируемой протеинкиназы (MAPK) и играющие важную роль в разнообразии клеточных ответов на стимуляцию IR и IGF-1R [41]. Несмотря на значительную гомологию белков семейства IRS и активацию многих сходных нижестоящих путей сигнализации, функциональные результаты передачи сигналов через IRS-1 или IRS-2 разнообразны, что подтверждено исследованиями на нокаутированных мышах [41].

Центральным событием является активация фосфатидилинозитол-3-киназой протеинкиназы В (AKT), через которую осуществляется регуляция метаболических эффектов инсулина и IGFs-регуляция поглощения глюкозы клетками для контроля ее гомеостаза, индукция гликолиза, негативная регуляция синтеза гликогена, липогенеза, глюконеогенеза, регуляция активности рапамицинового комплекса млекопитающих 1 (mTORC1), который опосредует регуляцию липогенеза и липолиза [37][41]. Через путь PI3K-AKT и mTORC1 реализуется и часть митогенных эффектов инсулина — дезактивация и деградация фактора транскрипции FOXO, отменяющая остановку клеточного цикла и активирующая подавление апоптоза клеток, индуцируется накопление в ядре клетки циклина D1, регулирующего фазовый переход клетки G1/S [37, 41]. В основном же митогенные эффекты (рост, пролиферация, дифференцировка, выживание клеток) опосредует активация сигнального пути RAS/MAPK/ERK. Множественные петли обратной связи и пересечения путей PI3K/AKT/mTOR и RAS/MAPK/ERK динамически регулируют сложные тканеспецифические эффекты на клеточный метаболизм и пролиферацию [37][41]. Однако в настоящее время стало очевидным, что классическая модель активации IGF-1R/IR с последующей инициацией единого сигнального ответа через пути PI3K/AKT и RAS/MAPK/ERK, независимо от состава рецепторного димера и воздействующего лиганда, сильно упрощена. Установлено, что на последующие сигнальные ответы влияют несколько факторов — аффинность связывания лиганд-рецептор, различия в связывании нижележащих белковых субстратов с рецептором в зависимости от комбинации лиганд–рецептор, различная кинетика интернализации рецептора и его рециклинга в зависимости от задействованной комбинации лиганд–рецептор, локализация рецептора в различных мембранных доменах [44].

В организме инсулин действует исключительно как системный гормон, регулируя метаболизм, при этом IGFs действуют не только системно, но и локально — аутокринным/паракринным образом [35][41]. Время их жизни, биодоступность в системе кровообращения и тканевой жидкости регулируется шестью типами высокоаффинных IGF-связывающих белков (IGFBP1-6), занимающих ключевое положение в регуляции передачи сигналов IGF, стабилизируя их экспрессию, ограничивая их взаимодействие с рецепторами, особенно с IR [45][46]. В частности, более 75% IGF-I в крови циркулирует в виде тройного комплекса в связи с IGFBP-3 и кислотостабильной субъединицей, что ограничивает циркуляцию в сосудистом русле. В результате диссоциации тройного комплекса образуется бинарный комплекс IGF с IGFBP, который покидает сосудистое русло, пересекая эндотелий для взаимодействия с рецептором в ткани-мишени. Менее 1% — свободный IGF, считающийся биоактивным [35]. Важным физиологическим механизмом высвобождения связанных IGF из комплекса с IGFBP являются неконсервативные линкерные домены, содержащие сайты для специфических протеаз, которые снижают аффинность связывания IGF. Соответствующие протеазы IGFBP тканеспецифичны и регулируют локально высвобождение IGF для связывания с рецептором. Несмотря на то что члены семейства IGFBP обладают значительной гомологией (N- и C-концевые домены), каждый из них имеет уникальные структурные особенности в виде функциональных мотивов, которые позволяют выполнять различные роли. Гены белков IGFBP имеют разные способы регуляции и паттерны экспрессии [35][46].

Исследования показывают, что IGFBP обычно связывают IGFs с равной или более высокой аффинностью, чем рецептор IGF-1R, и таким образом ингибируют передачу сигналов IGFs в тканях за счет секвестрации лигандов. Однако ряд IGFBP может усиливать действие IGFs, связываясь с протеогликанами на мембране клетки-мишени и/или компонентами внеклеточного матрикса, что приводит к локальной концентрации IGFs, его высвобождению при протеолитическом расщеплении IGFBP и связыванию IGFs с IGF-1R, усиливая передачу сигналов [35][47]. Помимо этого, было обнаружено, что некоторые IGFBP связываются со своими собственными рецепторами или транслоцируются в ядро некоторых типов клеток, где могут выполнять IGF-независимые функции, взаимодействуя с ядерными рецепторами, модулируя транскрипцию генов, пути других факторов роста [35][38]. Исследования потери функции на животных с нокаутом соответствующих генов хотя и дали относительно мало информации о физиологических функциях IGFBPs, но при этом ярко продемонстрировали высокую степень функциональной избыточности и/или наличие генетических компенсаторных механизмов в системе IGFBPs. Это предположение может объяснить отсутствие существенных фенотипов у мышей с нокаутом по отдельным IGFBP и обнаружение того, что животные могут выжить даже без трех из шести IGFBP. Существование множества IGFBP, скорее всего, способствует тонкой настройке контекстно-зависимой регуляции передачи сигналов в сети IGF в определенных типах клеток, а также при различных физиологических, стрессовых или патологических состояниях [38][47].

Таким образом, сигнальная система инсулина и IGFs представляет собой сложно организованную систему высокогомологичных лигандов, мембранных рецепторов и регуляторных связывающих белков, осуществляющую регуляцию различных сигнальных путей, оказывающих метаболические и митогенные эффекты. Точное функционирование этой системы обеспечивает рост и поддержание гомеостаза в физиологических и выживание организма в стрессовых условиях, а его нарушение приводит к развитию патологических состояний и усугубляет их тяжесть.

## ОСЬ IGF/IGFBP ПРИ САХАРНОМ ДИАБЕТЕ

Основным регулятором гомеостаза глюкозы является инсулин, но все больше данных подтверждает важную роль в этом процессе IGF-1, обладающего инсулиноподобным (метаболическим) действием, а также семейства IGFBP. Введение экзогенного IGF-1 снижает уровень глюкозы в сыворотке и улучшает чувствительность к инсулину у людей с СД2 и без него. IGFBP-1, взаимодействуя со свободным IGF-1 и связывая его, таким образом может регулировать уровень глюкозы. На основании этого была выдвинута гипотеза, что межиндивидуальные различия уровней эндогенного IGF-1 и IGFBP могут влиять на риск развития СД2 [42][48].

Исследования показали, что при формировании диабетической среды происходят значительные изменения в оси «гормон роста/его рецептор/IGF-1/IGFBP» [48]. В присутствии инсулина увеличивается экспрессия рецептора гормона роста в печени и активируется ось «гормон роста-рецепторы гормона роста», что приводит к стимуляции синтеза IGF-1 и подавлению продукции IGFBP-1, -2 и -3, которые ограничивают биодоступность IGF-1 в периферических тканях. Состояние нарушения толерантности к глюкозе и ранний СД2 характеризуются инсулинорезистентностью и гиперинсулинемией. Хроническая гиперинсулинемия связана с повышенным уровнем циркулирующего IGF-1 и сниженным уровнем IGFBP-1, -2 и -3. Таким образом, хроническая гиперинсулинемия способствует увеличению свободного (биодоступного) IGF-1 [49][50]. Однако есть сообщения о повышенном уровне IGFBP-1 у пациентов с СД2, возможно, это объясняется тем, что потеря чувствительности печени к инсулину и снижение уровня инсулина делают возможной неконтролируемую секрецию IGFBP-1 [42]. Собранные в обзоре M.S. Kim и соавт. результаты ряда исследований, в которых изучали уровни IGF-1 и IGFBP-1, -3 в сыворотке пациентов с СД, дают противоречивую информацию о связи изменений в оси IGF/IGFBP с клиническими переменными у пациентов с СД [42]. В работе Rajpathak S.N. и соавт. была установлена сильная связь случаев СД с исходными уровнями IGFBP-1, 2, 3 и свободного IGF-1, что свидетельствует о модулирующем эффекте оси IGF/IGFBP на риск развития СД. Результаты показали, что уровни IGFBP-1 и IGFBP-2 имеют сильную обратную связь с риском развития СД2 у женщин, тогда как уровень IGFBP-3 связан с повышенным риском, а уровень свободного IGF-1 при СД может варьироваться в зависимости от уровня инсулина. Таким образом, эти результаты показывают, что компоненты оси IGF могут играть определенную роль в патогенезе СД2 [51].

Однако ситуация осложняется тем, что IGFBP обладают значимой IGF-независимой активностью, включая потенциально полезные эффекты IGFBP-1 и IGFBP-2 на поглощение глюкозы [35][38]. IGF-1, напротив, может оказывать определенные неблагоприятные эффекты (например, способствовать пролиферации клеток) в дополнение к своей инсулиноподобной активности [41].

СД 1 типа — результат иммуноопосредованного разрушения β-клеток поджелудочной железы, приводящего к дефициту инсулина и повышению уровня глюкозы в крови. Это одно из наиболее распространенных хронических заболеваний у детей, возникает, как правило, в первые три десятилетия жизни. У пациентов с СД1 часто наблюдаются изменения в оси гормон роста/IGF-1, особенно в период полового созревания, когда в норме физиологические концентрации IGF-1 достигают пика. При портальной инсулинопении наблюдается гиперсекреция гормона роста гипофизом и снижение продукции IGF-1 в печени, его низкий уровень в крови. Возникает состояние резистентности печени к гормону роста [52]. В исследовании S.I. Chisalita и соавт. было показано, что у подростков с СД1 низкий уровень IGF-1 в крови повышался на начальном этапе лечения экзогенным инсулином, но с течением времени его содержание уменьшалось одновременно со снижением секреции эндогенного инсулина [53]. На уровень IGF-1 оказывают влияние как эффективность гликемического контроля, особенно в период полового созревания, так и продолжительность СД [52][54]. Однако у взрослых с СД1 низкий уровень IGF-1 не коррелирует с гликемическим контролем, но сохраняется зависимость от эндогенной секреции ­инсулина [55][56].

При дефиците инсулина снижение концентрации в крови IGF-1 сопровождается снижением концентрации IGFBP3 и ростом IGFBP-1. Изменения в профиле IGFBP приводят к изменению доступности IGF-1 при СД1, модулируя его биоактивность в крови и тканях [54][55][57]. Эти аномалии не только усугубляют гипергликемию у пациентов с СД1, но могут вносить вклад в патогенез диабетических осложнений [52][57][58].

Несмотря на очень большой объем проведенных исследований состояния оси IGF/IGFBP при СД обоих типов, в настоящее время все еще однозначно не определено, является ли нарушение регуляции данной оси первичным или вторичным по отношению к инициации и развитию СД.

## КОМПОНЕНТЫ ОСИ IGF/IGFBP ПРИ РАЗВИТИИ ЗЛОКАЧЕСТВЕННОЙ ОПУХОЛИ

В настоящее время установлена важная роль оси IGF/IGFBP в возникновении и развитии почти всех солидных злокачественных опухолей, в резистентности к химио- и лучевой терапии, таргетной терапии, причем стала очевидной значимость функциональных изменений всех компонентов оси [59–61]. Эпидемиологические данные выявили корреляцию высоких уровней IGF-1 в крови с повышенным риском рака [62]. Было показано, что люди с акромегалией, заболеванием, характеризующимся избытком гормона роста и IGF-1, имеют повышенный риск развития некоторых типов рака (колоректального, рака почки, молочной и щитовидной желез) [63][64]. При этом было обнаружено, что у людей с синдромом Ларона, заболеванием, характеризующимся первичной резистентностью к гормону роста и врожденным дефицитом IGF-1, злокачественные новообразования не развиваются [65][66].

Злокачественному фенотипу в значительной степени способствуют нарушения регуляции оси IGF/IGFBP — изменение биодоступности лигандов IGF-1,-2, сверхэкспрессия IGF1R, нарушение регуляции нижестоящих сигнальных эффекторов, при этом мутации рецептора встречаются редко. Надо отметить, что в большинстве солидных опухолей дисрегуляция оси сама по себе не является драйверной, а вторична по отношению к другому молекулярному событию, которое влияет на экспрессию лигандов или рецепторов. Изменения экспрессии компонентов оси IGF/IGFBP связывают с мутациями или аберрантной экспрессией регуляторов транскрипции, в частности в результате мутаций генов-супрессоров опухолей (BRCA1, WT1, VHL, TP53) происходит активация IGF1R. Кроме того, обнаружено, что р53 регулирует экспрессию и других компонентов системы IGF, включая INSR, IGF2 и IGFBP3 [67][68]. К аберрантной активации оси IGF приводит изменение биодоступности IGF, обусловленное в том числе сверхэкспрессией некоторых металлопротеиназ, специфически расщепляющих ряд белков IGFBP (IGFBP-3, -4) [68].

Кроме того, важная роль принадлежит изменениям в тесно связанной системе инсулина и его рецепторов. Ряд исследований показал, что гипергликемия сама по себе не может вызвать развитие опухоли в отсутствие гиперинсулинемии, что указывает на ключевую роль высокого уровня инсулина у пациентов с СД и ожирением в ­возникновении и прогрессировании злокачественных новообразований [69]. Хроническая гиперинсулинемия способствует увеличению уровня биодоступного IGF-1 (за счет увеличения уровня IGF-1 и снижения уровня IGFBP-1, -2, -3), а также увеличению образования гибридных рецепторов инсулина и IGF1R, тем самым увеличивая митогенный потенциал клетки [49][50]. IGF1R часто сверхэкспрессируется в злокачественно трансформированных клетках, т.к. обладает сильной антиапоптотической активностью, способствующей выживанию клеток. Повышенная экспрессия IGF1R в опухолях рассматривается как критическая адаптация, позволяющая уже трансформированным клеткам быстро пролиферировать и проходить через клеточный цикл [42]. В исследовании C. Xin и соавт. при РМЖ у женщин с СД2 была обнаружена более высокая экспрессия IGF1R по сравнению с пациентками без СД, а также более высокая экспрессия IGF1R в Her2-негативных опухолях молочной железы. Это свидетельствует о том, что IGF1R может быть альтернативным механизмом для пути передачи сигналов PI3K/AKT/mTOR при Her2-негативном РМЖ [70]. Похожие результаты были получены при обследовании пациентов с немелкоклеточным раком легкого с ранее существовавшим СД [71]. Связывание IGF1R с соответствующим лигандом (IGF-1, -2, инсулин) активирует несколько сигнальных путей, регулирующих метаболизм, пролиферацию, апоптоз, миграцию злокачественных клеток [72].

Тогда как IGF1R экспрессируется повсеместно и активирует пролиферативные и антиапоптотические пути, экспрессия изоформы IR-A сверхэкспрессирована на многих быстро делящихся трансформированных клетках [37]. Накопление гибридных рецепторов инсулина и IGF1R также характерно для опухолевых клеток [49][73]. IGF имеют значительно более высокое сродство к гибридам IR/IGF1R, чем инсулин, что приводит к оккупации IGF-1 и усилению пролиферации клеток, экспрессирующих оба рецептора, а основными нижестоящими эффекторами IR/IGF1R являются те же сигнальные пути PI3K/AKT и MAPK. В физиологических условиях (в отсутствие гиперинсулинемии) взаимодействие инсулина и IR-B носит фазовый характер и приводит к стимуляции PI3K/AKT/mTORC1, опосредуя анаболический каскад. При гиперинсулинемии (связанной с высокой экспрессией IR-A) и в опухолевых клетках (с высокой экспрессией IR-A и повышенным уровнем IGF-2, секретируемого опухолевыми клетками и/или опухолевой стромой) взаимодействие инсулина и/или IGF-2 с IR-A является устойчивым, и последующая активация RAS/MAPK/ERK опосредует митогенные эффекты инсулина, включая пролиферацию, выживаемость и миграцию клеток [39]. Дисбаланс между каскадами MAPK и PI3K приводит к нарушению метаболизма глюкозы/липидов в тканях-мишенях (печень, мышцы, жировая ткань) с активацией пролиферации клеток в других тканях [74]. Здесь следует добавить, что к настоящему времени выявлена индуцируемая IGF неканоническая функция IGF1R — рецептор после интернализации и эндоцитоза перемещается в ядро и действует как фактор транскрипции, связываясь с регуляторными областями ДНК [75].

Одним из важнейших аспектов регуляции оси IGF/IGFBP при развитии злокачественных новообразований является участие белков IGFBP. Значительное количество исследований выявило множество точек приложения IGFBP в этом процессе, несмотря на тот факт, что IGFBP обычно не мутируют при онкопатологии [45]. Учитывая участие IGFBP в ремоделировании тканей и пролиферации определенных типов клеток, неудивительно, что их физиологические действия могут координироваться опухолевыми клетками, чтобы обеспечивать рост опухоли [76]. Однако результаты многочисленных исследований в основном продемонстрировали то, что оказываемый IGFBP про- или противоопухолевый эффект очень сильно зависит от клеточного и тканевого контекста, т.е. относительной экспрессии рецепторов, особенностей состава внеклеточного матрикса, нижестоящих клеточных медиаторов, особенностей посттрансляционных модификаций молекул самих IGFBP (в частности, уровня фосфорилирования) и пр. Вся совокупность этих и многих других еще не изученных факторов является детерминирующей в определении эффекта. Преимущественно ингибирующее влияние на онкогенез было показано для IGFBP-1, что объяснялось высокой аффинностью к IGF-1 и предотвращением его связывания с рецептором IGF1R. Однако были выявлены положительные эффекты на пролиферацию, инвазию и миграцию опухолевых клеток через IGF-зависимые и IGF-независимые механизмы. В обзоре Y.W. Lin и соавт. представлены результаты многочисленных исследований роли IGFBP-1 в онкогенезе [77]. Считается, что противоречивые биологические эффекты IGFBP-1 связаны с его состоянием фосфорилирования: фосфорилирование IGFBP-1 повышает сродство к IGF, ингибируя его действие, а изоформа, стимулирующая действие IGF, не фосфорилирована. Тем не менее были обнаружены вероятно IGF-независимые эффекты: увеличение экспрессии IGFBP-1 в тканях РМЖ; установлено, что при раках желудочно-кишечного тракта IGFBP-1 может играть двойную роль в качестве супрессора опухолей и усилителя метастазирования, увеличение экспрессии IGFBP-1 в микроглиальных клетках, индуцированное секретируемым глиомой макрофагальным колониестимулирующим фактором, является важным медиатором в облегчении ангиогенеза опухоли [77]. Ангиогенезу в модели нейробластомы способствовала трансактивация гена VEGF посредством IGFBP-2, который, как оказалось, преимущественно способствует процессу онкогенеза. Было показано, что циркулирующие уровни IGFBP-2 коррелировали с опухолевыми маркерами агрессивности во многих видах рака [76]. Для IGFBP-3 был продемонстрирован в основном ингибирующий онкогенез эффект как через IGF-зависимые, так и IGF-независимые механизмы. Для IGFBP-5 были выявлены противоречивые эффекты на развитие опухоли, как и для IGFBP-1, связанные с контекстно-зависимыми различиями [45]. Исследования влияния IGFBP-6 предполагают, что он ингибирует рост опухоли посредством IGF-зависимых и IGF-независимых эффектов на пролиферацию, выживаемость и ангиогенез. Это проявлялось в его более низкой экспрессии в опухолевых клетках по сравнению с нормальными, включая наблюдения худшего прогноза при раке носоглотки и желудка при низких уровнях IGFBP-6 [76]. Напротив, независимые от IGF промиграционные эффекты IGFBP-6 могли быть проопухолевыми, поэтому роль IGFBP-6 в онкогенезе, как и остальных IGFBP, требует дальнейшего изучения.

## ЗАКЛЮЧЕНИЕ

В настоящее время в литературе представлены результаты огромного количества исследований системы IGF/IGFR/IGFBP в контексте двух патологий — СД и злокачественных новообразований. Эпидемиологические исследования дают обширный материал, свидетельствующий о наличии сильной положительной взаимосвязи между этими патологиями. До сих пор однозначно не установлена причинность этой связи. Однако предложен ряд вероятных биологических механизмов, которые объясняют эту связь через эффекты гипергликемии, гиперинсулинемии, воспаления на процесс онкогенеза. Гипергликемия в сочетании с гиперинсулинемией являются ключевыми факторами, увеличивающими агрессивность течения злокачественных опухолей.

Все больше данных свидетельствует о важной роли оси IGF/IGFBP не только в поддержании нормального метаболизма глюкозы и липидов, но и в патогенезе СД, злокачественных опухолей и взаимосвязи между ними. IGFs и их рецепторы, играя значительную роль в физиологии нормальных клеток, зачастую функционируют как решающий фактор выживания опухолевых клеток. Очень сложна роль IGFBP, участвующих в широком спектре клеточных процессов: благодаря IGF-зависимым и IGF-независимым механизмам эти компоненты оси IGF/IGFBP обеспечивают тонкую контекстно-зависимую регуляцию многих клеточных процессов, связанных с онкогенезом.

Несмотря на многолетнее глубокое изучение патогенеза СД обоих типов и злокачественных опухолей, молекулярные механизмы их взаимосвязи в сложной многоуровневой системе в настоящее время в значительной степени неясны. Внутренняя неоднородность СД и особенно онкологических заболеваний затрудняет проведение исследований, в результате чего все еще много вопросов остается без ответов. Каковы различия в механизмах влияния на онкогенез разных типов СД? Риск длительного воздействия высокого уровня инсулина изучен относительно мало и имеет непосредственное отношение к риску рака, связанному с продолжительностью диабета и использованием экзогенного инсулина. При измерении уровней инсулина, глюкозы и других аналитов в эпидемиологических исследованиях, как правило, оценивается только одна временная точка, что не дает представления об этапах течения патологии. В связи с этим необходимы дальнейшие исследования не только в клинических условиях, но и на экспериментальных моделях.

## ДОПОЛНИТЕЛЬНАЯ ИНФОРМАЦИЯ

Источник финансирования. Поисково-аналитическая работа проведена в рамках государственного задания: «Коморбидные заболевания как модификаторы течения злокачественного процесса».

Конфликт интересов. Авторы декларируют отсутствие явных и потенциальных конфликтов интересов, связанных с публикацией настоящей статьи.

Участие авторов. Все авторы внесли значимый вклад в написание статьи, прочли и одобрили финальный вариант рукописи.
